# Intergenerational effects of the pre-conception period on the number of services required by the daughter in dairy cows

**DOI:** 10.1371/journal.pone.0345080

**Published:** 2026-03-18

**Authors:** Abdelkader A. Ameur, Roger I. Cue, Marc-André Sirard

**Affiliations:** 1 Department of Animal Science, McGill University, Ste-Anne-de-Bellevue, Quebec, Canada; 2 Centre de recherche en reproduction, développement et santé intergénérationnelle, Faculté des sciences de l’Agriculture et de l’Alimentation, Département des Sciences Animales, Pavillon INAF, Université Laval, Québec, Québec, Canada; UPR: University of the Poonch Rawalakot, PAKISTAN

## Abstract

This study investigated how the metabolic status of dairy cow dams during the pre-conception period influences the fertility of their daughters. Using a large dataset from Lactanet Canada (2008–2023), we analyzed whether cumulative milk yield deviations—used as a proxy for energy balance—during the 40 days before conception were associated with the number of services required for conception across four daughter lactations. A linear mixed model incorporating milk yield deviations was employed. Results showed that daughters of high-yielding dams required more services (2.25) compared to those of low-yielding dams (2.15), suggesting impaired fertility associated with elevated maternal production. Additionally, daughters of multiparous dams required fewer services, indicating potential selection for fertility with increasing dam lactation number. Days in milk (DIM) at dam conception were positively associated with the number of services required by daughters in their first lactation. Overall, daughters in their first lactation required fewer services than those in subsequent lactations, reflecting a decline in reproductive efficiency with age. These findings highlight the influence of maternal metabolic status on offspring fertility, emphasizing the importance of managing dam energy balance during the preconception period to enhance herd reproductive performance.

## Introduction

Intergenerational inheritance refers to the transmission of epigenetic changes across immediate generations [1]. Laporta et al. [2] classified these effects as either intergenerational—transmitted from dam (F_0_) to daughter (F_1_)—or transgenerational, extending across multiple generations, such as to the granddaughter (F_2_), great-granddaughter (F_3_), and beyond. While heat stress has been extensively studied, the effects of other stressors, such as metabolic imbalance, remain insufficiently explored. Moreover, maternal characteristics like age and parity also influence progeny milk yield, with older dams often producing offspring with reduced milk yield [3–6]. Additionally, higher maternal milk production during gestation has been linked to lower milk yield in the offspring [3,4]. Swali and Wathes, [7] found first-parity dams’ offspring conceived faster, while Bafandeh et al. [8] reported higher fertility from offspring of late-parity dams. In addition, Walsh et al. [9] reported that daughters conceived while their dams were lactating had improved fertility compared with daughters conceived when their dams were not lactating, and Mossa et al. [10] showed maternal undernutrition early in gestation negatively impacts ovarian reserves in F_1_. Modern dairy cows, genetically driven to produce high milk yields, face significant health and reproductive challenges due to metabolic stress [11]. Postpartum negative energy balance in dairy cows, driven by the energy demands of milk production, leads to metabolic changes such as elevated NEFAs and BHB and reduced glucose. These changes disrupt the ovarian environment, impairing follicular function and oocyte quality, ultimately reducing fertility [12,13]. The preconception period is a critical window during which maternal metabolic status and nutritional balance significantly influence folliculogenesis, oocyte competence, and early embryonic development [14].

Previous studies assessing metabolic status in dairy cows have largely relied on blood sampling to measure key metabolites and biomarkers. Blood sampling, however, is an invasive method that has limited applicability on the farm. A possible alternative is to predict metabolic status of cows using readily accessible on-farm data, such as milk production parameters [15,16] and to assess the overall energy balance of cows at the herd level [17]. We hypothesize that the dam’s (F_0_) metabolic status, reflected by her milk yield during the periconceptional period—when the follicle develops, ovulates, and is fertilized—may influence the reproductive performance of her daughter (F_1_), as measured by the number of services required.

## Materials and methods

### Ethics statement

Approval from an animal welfare and use committee was not necessary for this study since the data were sourced from existing databases (Lactanet, Canada) that were compiled through standard milk recording practices.

### Retrospective assessment of records

To explore intergenerational associations between maternal metabolic status and daughter reproduction performance, a retrospective study was conducted using data from the Lactanet Canada consortium (2008–2023). The dataset included comprehensive records on animal identification, lactation performance, test-day, breeding events, and pedigrees, all linked via unique animal IDs. Key terms are defined as follows: dam (mother), daughter (female offspring), and animal (both sexes).

**Fig 1:**
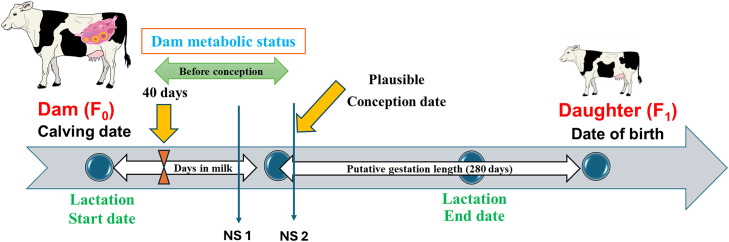
Identification of plausible daughter conception date. Images sourced from the CANEVAS website.

Fig 1 illustrates the overall concept of the study. The study links each dam (F_0_) to her daughters (F_1_) by matching the dam’s breeding (aka service) dates with the estimated conception date of the daughter (i.e., when the dam became pregnant with that daughter) using the putative gestation length and the recorded birth date and the recorded service dates to determine the most plausible service (i.e., conception) date.

**Fig 2:**
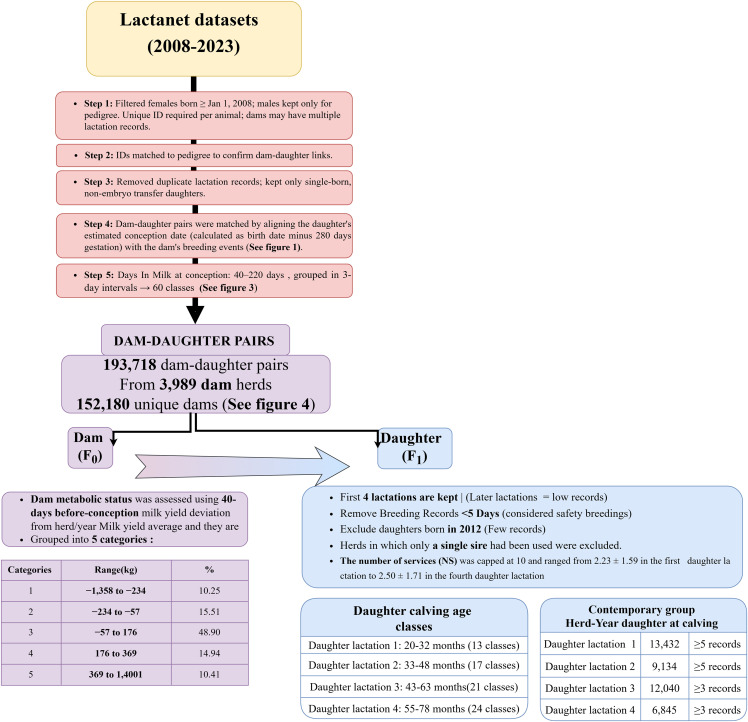
Diagram processing and filtering pipeline for dam–daughter pair analysis in Lactanet records.

The data processing workflow and filtering steps used to define the final dam-daughter dataset are presented in Fig 2. The initial dataset was filtered to include only females born after January 1, 2008. Duplicate lactation records were removed, and only single-born, non-embryo transfer daughters were retained. At this stage the data comprised 193,718 dam-daughter pairs from 3,989 dam herds and 152,180 unique dams.

To evaluate the dam’s metabolic status during the pre-conceptional period, milk yield over the 40 days preceding conception was calculated using test-day data, with linear interpolation applied when required. Dam milk yields were expressed as deviations from the corresponding herd–year–parity–days-in-milk (DIM) average calculated over the same 40-day period. For each herd-year of calving parity, and DIM, the average milk production of all dams of that parity during the preceding 40 days was calculated. Subsequently, for each dam included in the dam–daughter pairs, her own milk production over the same 40-day interval prior to conception was calculated, and the deviation from the corresponding herd–year–parity–DIM average was derived. For example, for a second-parity dam conceiving at 100 DIM, the average milk production of all second-parity cows in the same herd-year between 61 and 100 DIM was calculated to obtain the herd–year–parity–DIM average. The dam’s milk yield over the identical interval was then calculated, and her milk yield deviation was defined as the difference between her 40-day milk yield and the corresponding herd–year–parity–DIM average. These deviations were subsequently classified into one of five categories based on the quintiles of their distribution (Fig 2).

**Fig 3:**
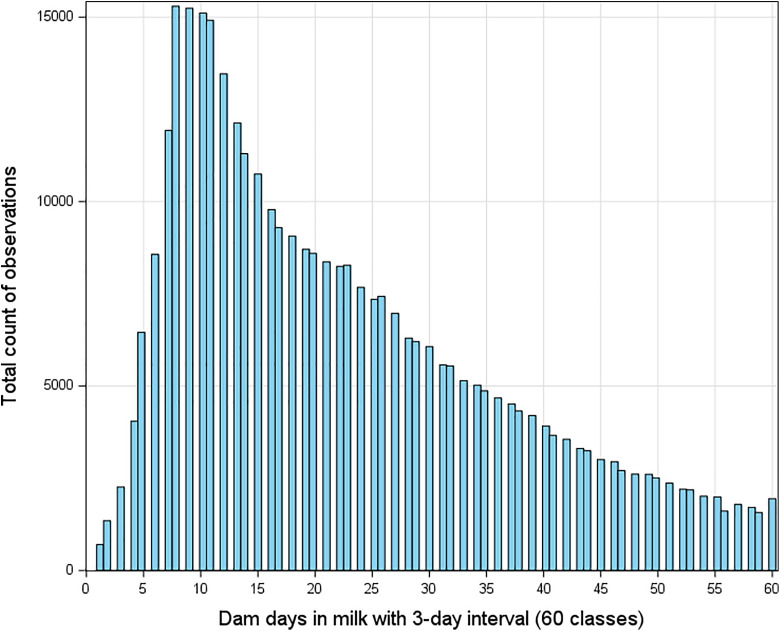
Histogram of dam days in milk 3 days category: frequency distribution by class.

Additionally, dam days in Milk (DIM) at conception—ranging from 40 to 220 days—were grouped into 3-day intervals (60 levels), this grouping increased the number of records per class, enhancing the precision of mean estimates and variance components (Fig 3).

Fertility traits are influenced by environmental, management, and physiological factors; among these, the number of services recorded for each daughter (F_1_)— our dependent variable—is a valuable indicator because it directly reflects reproductive efficiency and is less influenced by external conditions. Daughter records were edited so that only the first four lactations were retained, as later lactations had insufficient data. For each breeding event, if another breeding occurred within the following five days, the later event was removed because it was classified as a safety breeding. Daughters born in 2012 were excluded due to the small number of available records, and any herds in which only a single sire was used were also removed from the dataset. Contemporary groups (Herd/years of daughter calving) were required to include at least five records for lactations 1 and 2. Given the smaller group sizes in daughter lactations 3 and 4, the restriction for the number of animals in each contemporary group was reduced to a minimum of three records. Daughter age at calving was restricted to 20–32 months for the first lactation, 33–48 months for the second lactation, and corresponding age ranges for subsequent lactations (Fig 2). After all filtering and editing steps, the final dataset included 236,840 daughter (F_1_) records from 2,695 daughter herds and 97,220 unique dams (F_0_) across the first four lactations (Table 1).

**Table 1:** Final number of daughter (F_1_) lactation records, herds, and unique dams included in the analysis.

Daughter Lactation number	Total (F_1_) Records	Daughter (F_1_) herds	Unique Dams (F_0_)
**Lactation 1**	105,103	2,597	89,253
**Lactation 2**	64,732	2,282	56,769
**Lactation 3**	49,039	2,585	42,463
**Lactation 4**	21,733	2,117	19,522
**Total**	236,840 (From 115,161 dam-daughter-pairs Fig 4)	2,695	97,220

**Fig 4:**
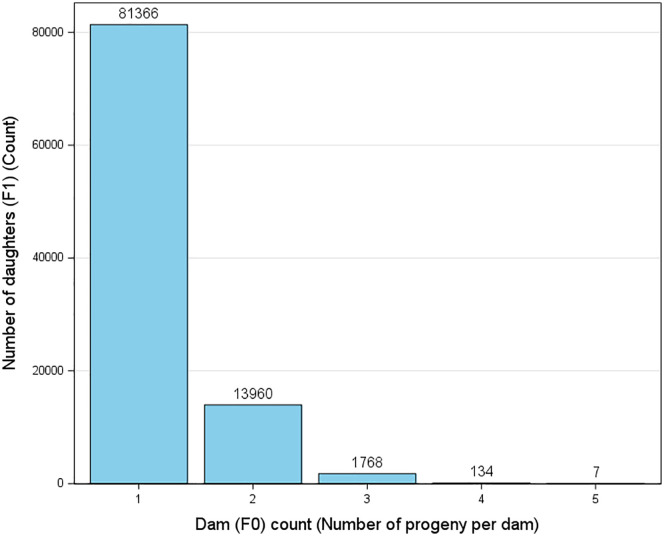
Distribution of number of daughters per dam. The total number of unique dams analyzed was 115,161 records.

### Statistical analysis

Statistical analyses were performed using a linear mixed model in SAS (v.9.4, SAS Institute Inc., Cary, NC, USA) to evaluate the associations of dam traits on the daughter’s NS. The PROC HPMIXED procedure was used. Separate models were applied for each individual daughter lactation. The final model used for the analyses was:

**Y**_**ijklmn**_ **= μ + HY**_**i**_ **+ AgeClv**_**j**_ **+ ClvMo**_**k**_ **+ DamDim**_**l**_ **+ DamParity**_**m**_ **+ DamMY**_**n**_ **+** β*DamBDate + e_**ijklmn**_

*Y*_*ijklmn*_ is daughter number of services observations; *μ* is overall population mean; *HY*_*i*_ is random effect of i^th^ of daughter herd-year at calving ~N(0,σ^2^_HY_)(see Fig 2); *AgeClv*_*j*_ is the fixed effect of j^th^ of daughter age at calving by month (see Fig 2); *ClvMo*_*k*_ is the fixed effect of k^th^ the daughter month of calving(January–December); *DamDim*_*l*_ is the fixed effect of l^th^ the dam days in milk (60 classes with equal interval 3 days); *DamParity*_*m*_ is the fixed effect of m^th^ dam parity (6 classes (1^st^, 2^nd^ and 3^rd^ daughter lactation) and 5 classes (4^th^ daughter lactation); *DamMY*_n_ is fixed effect of n^th^ dam milk yield deviate from herd/Year milk yield average (5 classes); *DamBDate* is the dam’s date of birth, modeled as a continuous covariate (to account for any potential of phenotypic, management and genetic trends over the period of dams’ dates of birth from 2008 to 2018); β is the coefficient for the regression of NS on dam birth date; e_*ijklmn*_ is the random residual term ~N(0,σ^2^_e_).

## Results and discussion

The number of services (NS) is an important metric for evaluating reproductive efficiency in dairy cattle. It reflects how many artificial inseminations are required on average to achieve a successful pregnancy. A lower NS value indicates higher reproductive efficiency. Looking at the variance components showed that the herd-year (HY) effect explained a small proportion of the total variability in the daughter NS across lactations. Specifically, HY accounted for 5.0% in lactation 1, 5.2% in lactation 2, 5.7% in lactation 3, and 5.8% in lactation 4. These results suggest that management and environmental factors shared within herds and years contribute modestly to reproductive outcomes.

The dam’s birth date was modeled as a continuous numeric variable and included in the linear regression model to account for phenotypic trends in the daughter’s NS from 2011 to 2021. The regression coefficients showed negative trends for all daughter lactations (Table 2), indicating a slight improvement in fertility. This aligns with efforts to integrate daughter fertility traits into selection indices [18], which are essential for improving reproductive performance despite the negative correlation between production and fertility [19].

**Table 2:** Regression coefficients (per day) (Estimate × 10 ⁻ ² and Standard Error × 10 ⁻ ^2^) for daughters across four lactations.

Daughter (F_1_) lactation number	Estimate (10 ^− 2^)	S.E. (10 ^− 2^)
Lactation 1	−0.017	0.0006
Lactation 2	−0.010	0.0010
Lactation 3	−0.011	0.0012
Lactation 4	−0.009	0.0021

### Daughter traits

#### Daughter age at calving.

The productive lifespan of a dairy cow, crucial for economic efficiency, is influenced by factors such as age at first calving, calving intervals, lactation duration, and the cow’s ability to continue into another lactation. Early maturity and consistent reproduction are key, as cows failing to conceive are often culled sooner. While these traits are important for reproductive success, they are heavily impacted by dairy management practices [20].

**Fig 5:**
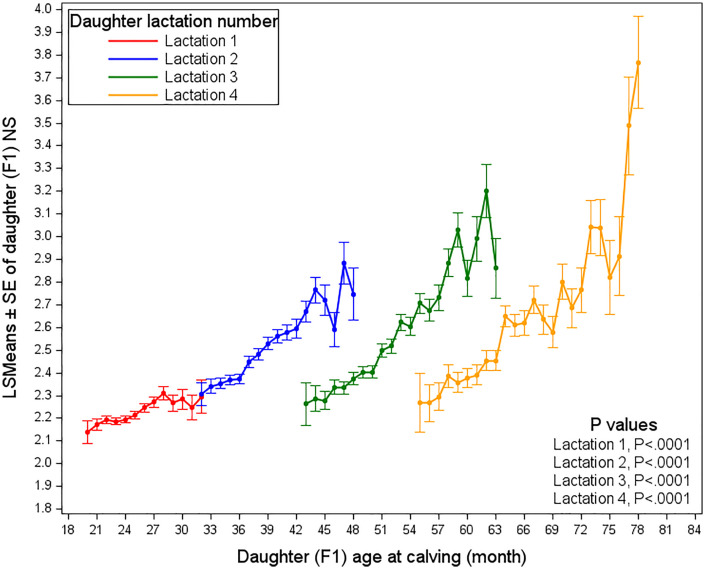
Least squares means of the number of services required by daughters, as affected by her age at calving and her lactation number.

Fig 5 illustrates the relationship between a daughter’s age at her calving and the number of services needed to conceive across her subsequent lactations (1–4). (Note: “Lactation” here referred to the cow’s own calving number, not the parity of her dam).

In daughter lactation 1 (1^st^ calving), the NS starts around 2.1 at 21 months of age and gradually increases to approximately 2.3 by 32 months, indicating that animals generally require fewer services and exhibit better fertility at younger ages at first calving. Daughter lactation 2 (2^nd^ calving) shows a marked increase in services between 32 and 48 months, reflecting both age-related fertility decline and possible autocorrelation, as animals with poorer inherent fertility tend to calve later and may retain this reduced fertility in subsequent lactations.

For daughter 3^rd^ lactation (3^rd^ calving), NS increased gradually after 54 months with greater variability, while daughter 4^th^ lactation (4^th^ calving) shows a more pronounced increase after 63 months, particularly around 75 months. In addition, an interesting pattern emerges when comparing daughter lactation 2 onward, the NS curves appear to be displaced by approximately 13 months, which is consistent with a typical calving interval. For example, cows calving at approximately 39 months in daughter lactation 2 and at 52 months in daughter lactation 3 exhibit very similar NS levels (2.53 services for daughter’s calving for second time at 39 months of age, versus 2.52 services for daughter’s calving for third time at 52 months of age), indicating that fertility stabilises from the second lactation onward. This apparent stability may partly reflect fertility-based culling, as cows with poorer reproductive performance in daughter lactation 2 are less likely to remain in the herd, resulting in slightly improved average fertility in daughter lactation 3. In contrast, fertility clearly deteriorates from daughter lactation 1–2, even in the presence of some fertility-based culling.

These findings were aligned with previous research, which suggests that while older cows may have increased reproductive experience and maturity, they face greater reproductive challenges, particularly at higher lactation number. For instance, older cows often experienced longer intervals between calving to first service and required more services due to physiological factors [21]. Moreover, the prevalence of reproductive disorders such as cystic ovarian disease in higher-lactation cows was extended the days open period and was increased the NS required for conception [22,23]. Younger cows showed better fertility and re-calving success, whereas older first-calving cows face longer calving intervals and lower conception rates [24]. Health complications and reproductive inefficiencies can also elevate the NS required for conception, particularly in older cows with higher lactation number [25]. These patterns reflect the reproductive challenges highlighted by López-Gatius [26], Royal et al. [27], and Lucy [28], who discuss declining reproductive efficiency with age and lactation number. Walsh et al. [29] and Wathes et al. [30] further emphasize the importance of managing fertility from early parities to ensure better lifetime reproductive performance, a finding supported by the favorable outcomes observed in daughter 1^st^ lactation in this study.

#### Daughter month of calving.

**Fig 6:**
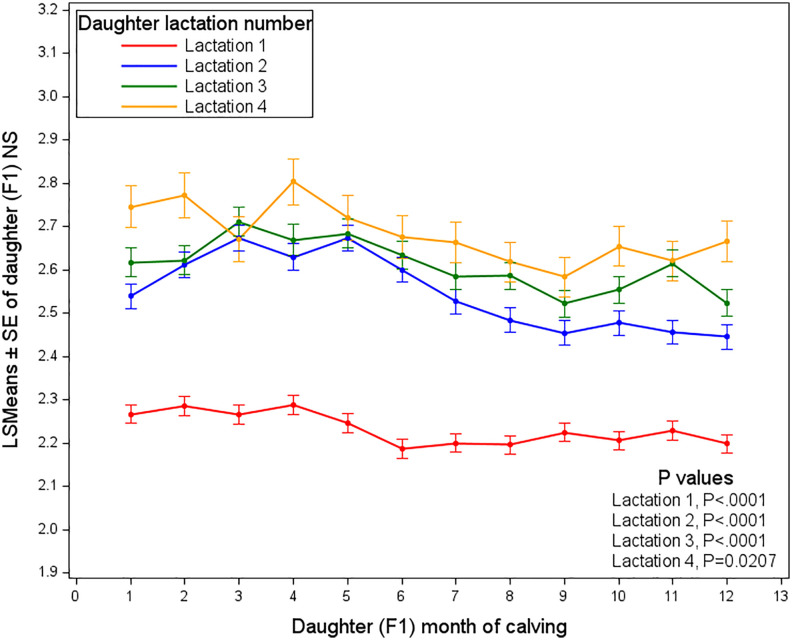
Least squares means of the number of services required by daughters, as affected by her month of calving and her lactation.

The relationship between the month of calving and the number of services required for conception across different daughter lactations in dairy cows is influenced by environmental conditions, reproductive health, and management practices. As illustrated in Fig 6, in daughter 1^st^ lactation, the NS remains stable throughout the year though a modest increase was observed during the early months (approximately January to March), with the number of services consistently around 2.2. In daughter second lactation, slightly higher NS required for conception were observed, peaking between March and May, followed by a gradual decrease, indicating some seasonal variation. In daughter lactation 3, a similar pattern was observed, with a slightly higher initial NS required for conception, peaking around months 4–6 before declining. Finally, in daughter lactation 4, the highest service demands were observed throughout the year, peaking early in month 2 and remaining elevated until month 6 before tapering off. These findings suggested that seasonal effects significantly impacted fertility, particularly in daughters of higher lactation numbers, which were more sensitive to calving month and tended to require more services when calving occurred in spring, as these cows were therefore bred during the summer months.

Seasonal impacts on fertility have been well documented, especially in high-yielding cows, which often experience longer intervals to first insemination and more open days during summer due to heat stress and reduced energy balance [31]. The warm season impairs reproductive performance through metabolic and endocrine disturbances, leading to lower oocyte quality and reduced conception success [32]. In contrast, Čítek et al. [23] reported improved reproductive outcomes for cows calving in cooler months (January to April) relative to those calving in summer. Similarly, Spring-calving cows showed higher reproductive efficiency than summer-calving cows, reflecting the adverse effects of heat stress on fertility [33,34]. From an evolutionary perspective, spring calving may also confer advantages by coinciding with more favorable environmental conditions. However, the observed seasonal effects may have been primarily influenced by conditions during the subsequent breeding period rather than by the calving month itself. For example, cows calving in early spring were often inseminated as temperatures rose, which may have negatively affected conception. This likely explained the increased NS observed during spring months, despite the general benefits associated with cooler-season calving. In year-round calving systems, these findings emphasized the importance of mitigating heat stress during summer breeding periods.

### Maternal effects

#### Dam days in milk.

**Fig 7:**
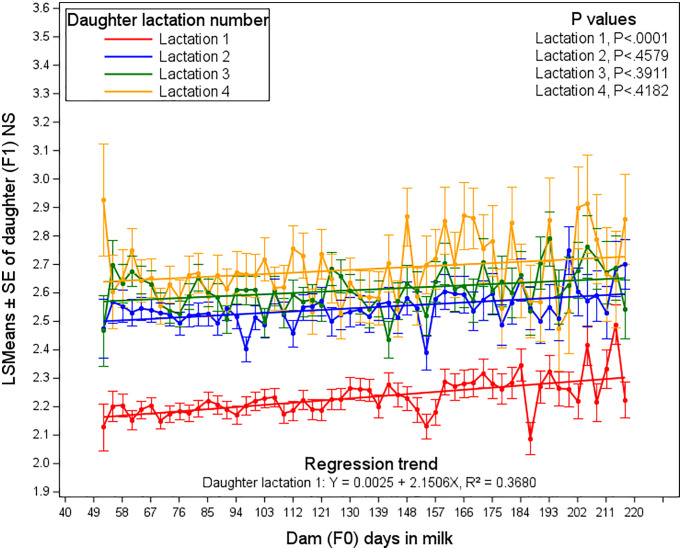
Least squares means of the number of services required by daughters, affected by dam days in milk (DIM) and daughter lactation.

Fig 7 illustrates the Least Squares Means (LSMEANS) estimates of the number of services required by daughters, as influenced by dam’s days in milk (DIM) at the time of daughter conception, across daughter lactations 1–4. DIM starts from 52 days rather than 40, due to the absence of milk yield deviation records during the first 12 days.

Throughout the dam lactation period from 52 to 220 days in milk, NS estimates showed clear differences among daughter lactations number. In fact, daughters in first lactation exhibited relatively low NS estimates, ranging from 2.15 to 2.40, indicating consistently better fertility performance than subsequent daughter lactations. Daughters in lactation 2 fluctuate between 2.30 and 2.50, while those in daughter lactation 3 range from 2.40 to 2.60. Daughters in lactation 4 consistently show the highest NS estimates, reaching up to 2.45 to 2.75 by day 220, reflecting ongoing reproductive challenges. To illustrate the overall pattern, we superimposed a linear regression line (for each daughter lactation number, 1–4) to show the overall trend across dam days in milk. The variability from one days in milk to another is broadly comparable across lactations.

The stability observed in lactation 1 daughters suggests that their fertility is less influenced by the physiological demands associated with increasing lactation number. In contrast, higher daughter’s lactations were showed more pronounced fluctuations, implying that as daughter advance through successive lactations, their physiological state may increasingly affect reproductive efficiency. No clear physiological explanation for these patterns has been established. This highlights the complex effect of a dam’s lactation stage (i.e., Dam DIM) on the reproductive efficiency of their daughters. First-lactation daughters differ from those in higher lactation in several ways: they are still growing, have no prior lactation experience, and face lower energy demands, which may enhance their reproductive performance compared to older cows that have experienced multiple pregnancies and lactation cycles [21,22,30]. Conversely, daughters of dams in early or late days in milk, especially from higher daughter lactation number, generally require more services to conceive, this effect was likely associated with the dam’s physiological status at different stages of her lactation, as metabolic and hormonal fluctuations during early or late lactation may impact the subsequent fertility of her offspring [25,29]. As daughters progress through their own lactations, the NS required for conception may increase, reflecting both intrinsic age-related changes such as reduced ovarian function and diminished oocyte quality and cumulative lactation-related stress. Additionally, the dam’s DIM at conception reflects her metabolic demands and lactation status, which can shape the daughter’s future fertility. Supporting this, Harati et al. [35] reported that daughters of high-producing dams typically further along in lactation and experiencing greater metabolic strain exhibited poorer reproductive performance, including longer intervals to conception and reduced fertility, compared with daughters of lower-producing dams.

#### Dam parity.

**Fig 8:**
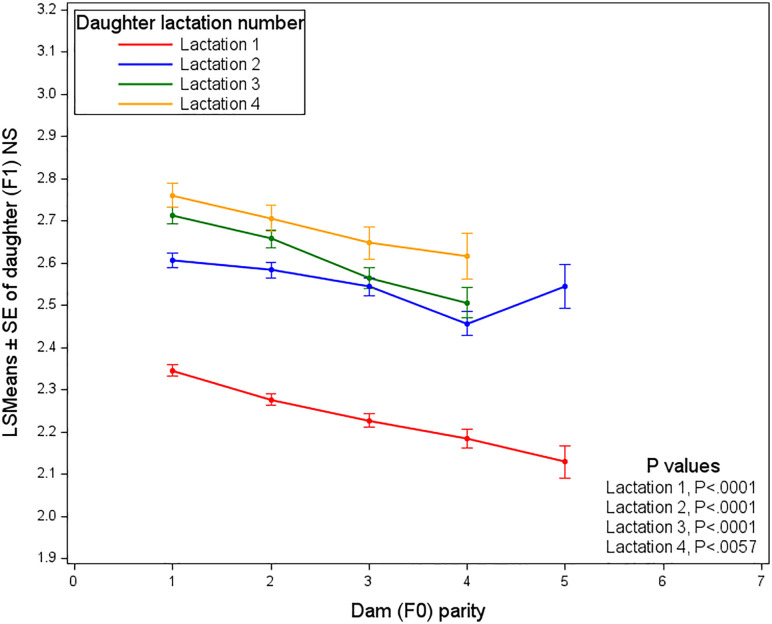
Least squares means of the number of services required by daughters, affected by dam parity and daughter lactation number.

Fig 8 presents the Least Squares Means (LSMEANS) estimates of the number of services required by daughters as influenced by the parity of the dam, analyzed across different daughter lactations (1^st^ to 4^th^). As the dam’s parity increased, the NS needed for conception generally decreased. Daughters of more experienced dams (higher parities) were found to require fewer services. This trend was most pronounced in first lactation daughters, where a clear decline in NS was observed as dam parity increased. For daughters in their 2^nd^ to 4^th^ lactation, the decrease was more gradual, though it remained noticeable. Overall, higher dam parity was linked to better daughter fertility and fewer required services.

Dam parity consistently influences first-lactation performance of their daughter. Studies by Fuerst-Waltl et al. [36], González-Recio et al. [5], and Astiz et al. [37] all reported that heifers born to younger dams generally exhibited superior productive performance during their first parity, particularly in higher milk yield, whereas effects on fertility and somatic cell score were negligible or inconsistent across studies. However, it was suggested by our findings that as dams progress through multiple parities, their daughters may have benefited from enhanced reproductive efficiency, requiring fewer services to conceive. This improvement is attributed to better maternal care, a more favorable uterine environment, or other physiological advantages associated with mature dams. Additionally, selection pressure and culling practices over successive lactations may have improved the overall reproductive profile of the herd. However, given the low repeatability of fertility traits, the consistency of reproductive performance across lactations and consequently the direct impact on daughter fertility may be limited.

In this context, Akbarinejad et al. [38] found that younger dams, which were still growing while reproducing, had increased nutritional demands to support both their own development and that of the fetus. These competing requirements, differing from those of mature dams, were shown to adversely affect offspring development and reproductive potential [39]. Banos et al. [3] similarly reported that offspring from younger dams had lower non-return rates and required more inseminations to conceive compared to offspring from older dams. Additionally, several studies focusing on nulliparous dams reported reduced antral follicle count (AFC), delayed days to first service postpartum, prolonged first service to conception intervals, increased NS per conception, and extended calving-to-conception intervals in their offspring [8,9,35,38,40–42]. Thomson et al. [43] recently observed that offspring born to nulliparous dams exhibited lower anti-Müllerian hormone (AMH) concentrations and greater average daily gain from pre-conception to early gestation

#### Dam milk yield.

**Fig 9:**
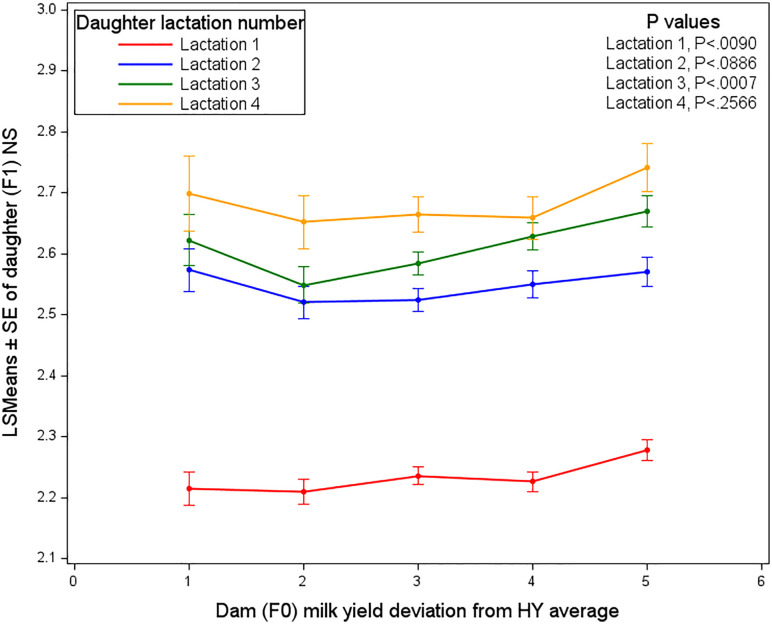
Least squares means of the number of services required by daughters, affected by dam milk yield deviation and daughter lactation number.

Fig 9 presents the Least Squares Means (LSMEANS ± SE) of the number of services required by daughters (F_1_) in relation to their dams’ (F_0_) milk yield deviation from the herd–year (HY) average, stratified by daughter lactations (1–4). Statistically significant associations were found only for daughter lactations 1 and 3.

In daughter lactation 1, daughters of lower-yielding dams (Classes 1–2) required fewer services to conceive compared with daughters of higher-yielding dams (Classes 4–5). The NS increased progressively from approximately 2.21 in Class 1 to 2.28 in Class 5, indicating a decline in reproductive efficiency with increasing positive deviation in dam milk yield.

For daughter lactations 2 and 3, the NS was observed to remain relatively stable across most dam milk-yield deviation classes; however, daughters originating from dams in the highest milk-yield deviation class (Class 5) were consistently required to receive more inseminations to achieve conception. This pattern indicates that exceptionally high relative maternal milk production, rather than a linear increase across yield classes, is associated with reduced reproductive performance in daughters beyond first lactation. In daughter lactation 4, although the highest overall NS was observed across all milk-yield deviation classes, numerically higher values were again recorded for Class 5, but differences among the five classes were not found to be statistically significant. This indicated that while extremely high dam milk yield may have been detrimental to daughter fertility across lactations, factors accumulating over successive lactations were likely to have played a more prominent role in determining reproductive efficiency at later daughter lactations, particularly given that age at calving was accounted for in the model.

Prenatal exposure to maternal metabolic stress can negatively impact offspring health. Therefore, the elevated maternal milk production is a significant source of metabolic stress in high dairy cows [44,45]. In our study, the dam milk yield deviations from 40 days prior to conception up to conception affected the NS required in daughter first lactation, indicating a trade-off between dam milk production and daughter reproductive efficiency. In subsequent lactations, this effect decreased, which may be due to selection or improved management practices. The findings support the hypothesis that dams with higher-than-average milk yields (classes 4 and 5) may experience stress that could lead to reduced reproductive efficiency in their daughters, as evidenced by the increased NS required. This is further supported by a recent study, which revealed that offspring of high-producing dams have a longer interval from calving to conception [35], suggesting that high milk production may negatively impact reproductive performance. Additionally, daughters of igh-producing dams tend to exhibit higher milk yields, but poorer metabolic health early postpartum, which may further impair their reproductive [8,46–50]. Moreover, Roche et al. [51] emphasized that periparturient management, including the cow’s nutritional status, is crucial for optimizing reproductive performance.

High maternal milk production can lead to increased services per conception, thereby affecting overall reproductive efficiency. This intricate relationship highlights the importance of managing maternal health and production traits to enhance reproductive outcomes in dairy herds. Recent findings by López-Catalina et al. [52] further suggest that calves gestated by lactating dams exhibit distinct methylation profiles compared to those gestated by nonlactating heifers and Wang et al. [53] later confirmed that periconceptional milk yield predicted daughter metabolic dysregulation, including elevated blood glucose and reduced milk fat synthesis. This potential status effect warrants further investigation, particularly to understand the underlying mechanisms, such as metabolic, hormonal, or epigenetic changes, which could contribute to this relationship.

Fertility in dairy cattle is a vital component of sustainable livestock production, significantly impacting herd productivity and economic viability. Recent evidence suggests that fertility decline in dairy herds has slowed or reversed in some areas due to genomic selection improving reproductive traits or the use of hormonal estrus synchronisation protocols [54]. However, some challenges are often linked to various factors, including metabolic status, which occur when an animal’s energy demand exceeds its energy intake. Such status can have profound implications not only on the health and productivity of the dam, but also on the reproductive performance of her offspring, leading to intergenerational effects that can influence the reproductive efficiency of future generations. The preconception period, the time surrounding conception, including the stages of folliculogenesis and early embryo development, is particularly crucial for ensuring successful reproduction in dairy cattle. Cumulative milk yield has been identified as an important proxy for assessing metabolic status in dairy cows, as deviations from the herd average provide insight into the dam’s overall energy status. Our findings emphasize the relationship between maternal milk production and reproductive efficiency in offspring, reinforcing the importance of managing metabolic status during critical periods to improve fertility outcomes in dairy cattle. This study aims to deepen the understanding of these dynamics and their implications for sustainable dairy production.

## Supporting information

S1 File STROBE-checklist-v4-combined-PlosMedicine.docx. (DOCX)
