# Supplementary material for: Intergenerational effects of the pre-conception period on the number of services required by the daughter in dairy cows
Source: PLoS One. 2026 Mar 18;21(3):e0345080. doi: 10.1371/journal.pone.0345080 (PMC12998810; doi:10.1371/journal.pone.0345080)
Supplement: S1 File — (DOCX) [file pone.0345080.s001.docx]

STROBE Statement—checklist of items that should be included in reports of observational studies

|  | Item No. | Recommendation | Page  No. | Relevant text from manuscript |
| --- | --- | --- | --- | --- |
| **Title and abstract** | 1 | (*a*) Indicate the study’s design with a commonly used term in the title or the abstract | 1 | Title and Abstract clearly describe a *retrospective study* based on Lactanet Canada data (2008–2023) |
|  |  | (*b*) Provide in the abstract an informative and balanced summary of what was done and what was found | 1 | Abstract describes data sources, methods (linear mixed model), findings (maternal milk yield deviations affect daughter fertility), and conclusion (maternal energy balance matters). |
| Introduction | | | |  |
| Background/rationale | 2 | Explain the scientific background and rationale for the investigation being reported | 1-2 | Introduction explains intergenerational inheritance, effects of maternal metabolic status, and knowledge gaps. |
| Objectives | 3 | State specific objectives, including any prespecified hypotheses | 2 | Hypothesis: dam metabolic status during pre-conception (milk yield deviations) affects daughter fertility (number of services). |
| Methods | | | |  |
| Study design | 4 | Present key elements of study design early in the paper | 3 | “A retrospective study was conducted using data from the Lactanet Canada consortium (2008–2023).” |
| Setting | 5 | Describe the setting, locations, and relevant dates, including periods of recruitment, exposure, follow-up, and data collection | 3 | Setting: Dairy herds across Quebec; data from 2008–2023; variables include milk yield, breeding, lactation, pedigree |
| Participants | 6 | (*a*) *Cohort study*—Give the eligibility criteria, and the sources and methods of selection of participants. Describe methods of follow-up  *Case-control study*—Give the eligibility criteria, and the sources and methods of case ascertainment and control selection. Give the rationale for the choice of cases and controls  *Cross-sectional study*—Give the eligibility criteria, and the sources and methods of selection of participants | 3-4 | Included females born after Jan 1, 2008; single-born, non-embryo transfer daughters; duplicates removed. |
|  |  | (*b*) *Cohort study*—For matched studies, give matching criteria and number of exposed and unexposed  *Case-control study*—For matched studies, give matching criteria and the number of controls per case | N/A | no matching design used |
| Variables | 7 | Clearly define all outcomes, exposures, predictors, potential confounders, and effect modifiers. Give diagnostic criteria, if applicable | 4 | Outcome: number of services per conception (NS). Exposures: dam milk yield deviation, DIM, Dam parity. herd-year, daughter age, month of calving. |
| Data sources/ measurement | 8* | For each variable of interest, give sources of data and details of methods of assessment (measurement). Describe comparability of assessment methods if there is more than one group | *3-4* | Data from Lactanet (test-day milk yields, breeding records, pedigrees). Milk yield deviations calculated as differences from herd average. |
| Bias | 9 | Describe any efforts to address potential sources of bias | 3-4    5 | For data editing and validation  For the statistical model including factors which thought might be cofounding effects. |
| Study size | 10 | Explain how the study size was arrived at | 4 | Used all available qualified data after filtering: 193,718 dam–daughter pairs from 3,989 herds. |

Continued on next page

| Quantitative variables | 11 | Explain how quantitative variables were handled in the analyses. If applicable, describe which groupings were chosen and why | 4 | Quantitative traits grouped (e.g., DIM in 60 classes, milk yield in 5 deviation classes) to stabilize variance. |
| --- | --- | --- | --- | --- |
| Statistical methods | 12 | (*a*) Describe all statistical methods, including those used to control for confounding | 4 | Linear mixed model (PROC HPMIXED, SAS v9.4) with herd-year random effect and various fixed factors (e.g., dam DIM, parity, milk yield deviation). |
|  |  | (*b*) Describe any methods used to examine subgroups and interactions | 4-5 | Separate models run for each daughter parity to explore subgroup differences |
|  |  | (*c*) Explain how missing data were addressed | 3-4 | Records with missing or inconsistent data removed during filtering. |
|  |  | (*d*) *Cohort study*—If applicable, explain how loss to follow-up was addressed  *Case-control study*—If applicable, explain how matching of cases and controls was addressed  *Cross-sectional study*—If applicable, describe analytical methods taking account of sampling strategy | N/A |  |
|  |  | (*e*) Describe any sensitivity analyses | N/A |  |
| Results | | | | |
| Participants | 13* | (a) Report numbers of individuals at each stage of study—eg numbers potentially eligible, examined for eligibility, confirmed eligible, included in the study, completing follow-up, and analysed | 4 | After filtering: 193,718 dam–daughter pairs,  First parity daughters: 109,756;  Second Parity daughters: 67,132;  Third Parity daughters: 49,039;  Fourth Parity daughters:21,733. |
|  |  | (b) Give reasons for non-participation at each stage | 3-4 | Exclusion of duplicates, embryo-transfer, and incomplete records. |
|  |  | (c) Consider use of a flow diagram | 3-4 | Figure 1 presents data processing workflow. |
| Descriptive data | 14* | (a) Give characteristics of study participants (eg demographic, clinical, social) and information on exposures and potential confounders | 5 | Described dam and daughter traits (age, parity, milk yield, DIM, month of calving) in Results and Figures 5–9. |
|  |  | (b) Indicate number of participants with missing data for each variable of interest | N/A |  |
|  |  | (c) *Cohort study*—Summarise follow-up time (eg, average and total amount) | N/A |  |
| Outcome data | 15* | *Cohort study*—Report numbers of outcome events or summary measures over time | *5* | Number of services (NS) across 4 daughter parities |
|  |  | *Case-control study—*Report numbers in each exposure category, or summary measures of exposure |  |  |
|  |  | *Cross-sectional study—*Report numbers of outcome events or summary measures |  |  |
| Main results | 16 | (*a*) Give unadjusted estimates and, if applicable, confounder-adjusted estimates and their precision (eg, 95% confidence interval). Make clear which confounders were adjusted for and why they were included | 3 | Unadjusted averages of Number of services ranged from 2.23 ± 1.59 (1^st^ parity) to 2.50 ± 1.71(4^th^ parity) |
|  |  | (*b*) Report category boundaries when continuous variables were categorized | 4 | DIM (60 3-day intervals), milk yield deviation (5 classes). |
|  |  | (*c*) If relevant, consider translating estimates of relative risk into absolute risk for a meaningful time period | N/A |  |

Continued on next page

| Other analyses | 17 | Report other analyses done—eg analyses of subgroups and interactions, and sensitivity analyses | 4 | Dam days milk is initially tested as 181 days classes and we concluded that 60, 3-day classes is more parsimonious and adequate.  Preliminary analysis for the dam milk yield deviation indicate that 5 classes was more informative than 3 classes. |
| --- | --- | --- | --- | --- |
| Discussion | | | | |
| Key results | 18 | Summarise key results with reference to study objectives | 12 | High milking yield dams have daughter with more insemination required |
| Limitations | 19 | Discuss limitations of the study, taking into account sources of potential bias or imprecision. Discuss both direction and magnitude of any potential bias | 12-13 | Indirect measure for the metabolic load on the dam preconception |
| Interpretation | 20 | Give a cautious overall interpretation of results considering objectives, limitations, multiplicity of analyses, results from similar studies, and other relevant evidence | 9-14 | About the Dam effect (DIM, Parity and relative milk yield) on the daughter fertility |
| Generalisability | 21 | Discuss the generalisability (external validity) of the study results | 11 | Fuerst-Waltl et al. , González-Recio et al. , and Astiz et al. these authors concluded the dam parity effect , where their results are consistent with my results. |
| Other information | |  | | |
| Funding | 22 | Give the source of funding and the role of the funders for the present study and, if applicable, for the original study on which the present article is based | 15 | Funded by the Resilient Dairy Genome Project (Genome Canada, Genome Quebec, etc.); funders had no role in analysis or interpretation. |

*Give information separately for cases and controls in case-control studies and, if applicable, for exposed and unexposed groups in cohort and cross-sectional studies.

**Note:** An Explanation and Elaboration article discusses each checklist item and gives methodological background and published examples of transparent reporting. The STROBE checklist is best used in conjunction with this article (freely available on the Web sites of PLoS Medicine at http://www.plosmedicine.org/, Annals of Internal Medicine at http://www.annals.org/, and Epidemiology at http://www.epidem.com/). Information on the STROBE Initiative is available at www.strobe-statement.org.
